# Type 2 diabetes mellitus and post-colonoscopy colorectal cancer: clinical and molecular characteristics and survival

**DOI:** 10.1007/s10552-024-01861-9

**Published:** 2024-03-14

**Authors:** Mette L. Boysen, Frederikke S. Troelsen, Henrik T. Sørensen, Rune Erichsen

**Affiliations:** 1 Department of Surgery, Gødstrup Regional Hospital, 7400 Herning, Denmark; 2 Department of Clinical Epidemiology, Aarhus University Hospital, Olof Palmes Allé 43-45, 8200 Aarhus N, Denmark; 3 Department of Surgery, Randers Regional Hospital, 8930 Randers, Denmark

**Keywords:** Colorectal cancer, Colonoscopy, Type 2 diabetes mellitus, Cohort, Population-based

## Abstract

**Purpose:**

Studies suggest that patients with type two diabetes mellitus (T2D) may be at increased risk of post-colonoscopy colorectal cancer (PCCRC). We investigated clinical and molecular characteristics and survival of T2D patients with PCCRC to elucidate how T2D-related PCCRC may arise.

**Methods:**

We identified T2D patients with colorectal cancer (CRC) from 1995 to 2015 and computed prevalence ratios (PRs) comparing clinical and molecular characteristics of CRC in T2D patients with PCCRC *vs.* in T2D patients with colonoscopy-detected CRC (dCRC). We also followed T2D patients from the diagnosis of PCCRC/dCRC until death, emigration, or study end and compared mortality using Cox-proportional hazards regression models adjusted for sex, age, year of CRC diagnosis, and CRC stage.

**Results:**

Compared with dCRC, PCCRC was associated with a higher prevalence of proximal CRCs (54% vs*.* 40%; PR: 1.43, 95% confidence interval [CI] 1.27–1.62) in T2D patients. We found no difference between PCCRC *vs.* dCRC for CRC stage, histology, and mismatch repair status. The proportion of CRCs that could be categorized as PCCRC decreased over time. Within one year after CRC, 63% of PCCRC vs. 78% of dCRC patients were alive (hazard ratio [HR] 1.85 [95% CI 1.47–2.31]). Within five years after CRC, 44% of PCCRC vs. 54% of dCRC patients were still alive (HR 1.44 [95% CI 1.11–1.87]).

**Conclusion:**

The increased prevalence of proximally located PCCRCs and the poorer survival may suggest overlooked colorectal lesions as a predominant explanation for T2D-related PCCRC, although altered tumor progression cannot be ruled out.

**Supplementary Information:**

The online version contains supplementary material available at 10.1007/s10552-024-01861-9.

## Introduction

According to the World Endoscopy Organization (WEO), post-colonoscopy colorectal cancer (PCCRC) is defined as colorectal cancer (CRC) diagnosed >6 months and up to 36 months after a colonoscopy negative for CRC [1]. It is assumed that the majority of PCCRCs occur due to precancerous lesions that were missed or incompletely removed during the colonoscopy procedure [1]. Only a small proportion is thought to arise from different tumor biology underlying CRC development [1–3]. Existing literature indicates an increased proportion of PCCRC in patients with specific diseases such as diverticular disease and inflammatory bowel disease, with PCCRCs accounting for up to 50% of all CRCs diagnosed among these patients [1, 4, 5]. In addition, three studies have suggested an association between type two diabetes (T2D) and PCCRC [6–8]. Hence Laish *et al.* reported that 31% of all PCCRC patients had prevalent diabetes mellitus, while Suceveanu *at al.* found PCCRC and adenomas to be more frequent among patients with T2D than among patients without T2D [6, 7]. Finally, Troelsen *et al.* suggested an increased T2D-related risk of PCCRC [8]. T2D patients may achieve poorer bowel preparation for colonoscopy than non-T2D patients due to functional impairment of gastrointestinal motility. While such impairment could complicate colonoscopy and increase the risk of overlooking CRC and its precursors, evidence remains limited [9, 10]. Existing literature also indicates that the prevalence of proximally located polyps is higher in T2D patients, a localization associated with an increased risk of missed polyps and thus PCCRC [11]. At the same time, the consequences of long-term T2D (*e.g.*, hyperinsulinemia, hyperglycemia, and microbiota changes) may affect the carcinogenenic processes leading to CRC growth in the interval between two colonoscopies [12, 13]. These findings combined could lead to a different ratio between T2D-related PCCRCs that arise through missed precursors and different or cancer development than for non-diabetic patients, but evidence is missing. The issue is compounded by the increasing global incidence of T2D [14, 15] and the introduction of CRC screening programs with the same inclusion criteria for T2D patients and non-diabetic patients. We therefore conducted the present study to examine characteristics and survival of T2D-related PCCRC to elucidate potential explanations for T2D-related PCCRC.

## Methods

### Setting and data sources

This nationwide study of T2D prevalence and prognosis was designed to use prospectively collected data from Danish national health databases during the January 1, 1995–December 31, 2015 period. The study was conducted within the setting of universal, tax-funded health care provided to all legal residents of Denmark by the National Health Service [16]. All persons residing in Denmark are registered in the Danish Civil Registration System (CRS) and assigned a unique personal identification number, which enables accurate and individual-level data linkage among all Danish health and administrative registries [16–18]. All registries and codes used in the analyses are described in Supplementary Tables 6-8.

The study was reported to the Danish Data Protection Agency by Aarhus University (record No. 2016-051-000001/1671).

### Colorectal cancer patients

Patients with a first-time CRC diagnosis were identified from the Danish Cancer Registry (DCR). The DCR also provided information on CRC diagnosis date, location, and stage at diagnosis. CRCs were categorized using the following anatomic locations: proximal to the splenic flexure, distal to the splenic flexure, rectal, or unspecified/more than one site. CRCs also were categorized by stage at diagnosis according to the TNM classification: localized, regional, metastatic, or unknown.

Information on tumor histology at diagnosis and mismatch repair status (MMR) (when available) was obtained from the Danish National Pathology Registry (DPR). We categorized CRC histology as adenocarcinoma, polyp adenocarcinoma (*i.e.*, invasive growth within an endoscopically well-defined polyp), mucinous carcinoma, and signet cell cancer. CRCs lacking a histological diagnosis in the DPR were categorized as “not histologically verified”. CRCs with an available test result for MMR status (N=61.8%) demonstrating nuclear absence of at least one of four MMR proteins (MLH1, MSH2, MSH6, or PMS2) were categorized as MMR-deficient. CRCs with a test demonstrating normal MMR status were categorized as MMR-proficient.

In addition, we categorized CRCs according to surgeries performed, based on surgical codes from the Danish National Patient Registry (DNPR) recorded within 90 days following a CRC diagnosis.

### Detected and post-colonoscopy colorectal cancers

We linked the CRC cohort to the DNPR to obtain information on colonoscopies performed within three years before the CRC diagnosis. By design, the study excluded all CRCs diagnosed in patients who did not undergo a colonoscopy during this prior three-year period. All included CRCs were categorized as either PCCRCs or CRC detected during colonoscopy (dCRCs). We defined PCCRCs in accordance with World Endoscopy (WEO) recommendations, *i.e*., CRC diagnosed >6 to 36 months after a colonoscopy that found no CRC [1] and dCRCs as CRC diagnosed within 6 months after a colonoscopy.

### Assessment of diabetes mellitus

We identified PCCRC and dCRC patients who also had a T2D diagnosis from the DNPR and the Danish National Health Service Prescription Database (DNHSPD). All patients who redeemed a prescription for a glucose-lowering drug and/or had a diagnosis of diabetes mellitus recorded before or within 90 days after the colonoscopy that failed to detect the CRC/precancerous lesion (for PCCRCs) or that detected the CRC (for dCRCs) were considered to be T2D patients. According to a previously reported algorithm [19], patients younger than 30 years of age, who had a registered diagnosis of diabetes or redeemed a prescription for a glucose-lowering drug before or within 90 days of the colonoscopy that either failed to detect or detected the CRC were considered to be have type 1 diabetes and were excluded from the study. We categorized T2D patients according to T2D duration, measured in years from first-time prescription of a glucose-lowering drug or a first-time T2D diagnosis until CRC diagnosis (< 1 year, 1–5 years, 6–10 years, and > 10 years). Patients without T2D were excluded.

### Assessment of covariates

We collected data from the DPR on colorectal polyps detected before the diagnosis of CRC. We included sessile serrate lesions and conventional adenomas. If no information concerning polyps was found in the DPR, we searched the DNPR using codes for endoscopically performed polypectomies recorded before the CRC diagnosis. We collected information on comorbidities from all records available in the DNPR from 1977 until the date of CRC diagnosis. To characterize the study cohort, we obtained discharge diagnoses of chronic obstructive pulmonary disease, cardiovascular diseases including atrial fibrillation/flutter, renal disease, alcohol-related diseases (*i.e.,* alcohol-related psychosis, alcoholism, alcohol intoxication, chronic alcoholic pancreatitis, alcoholic liver cirrhosis, steatosis, brain degenerative changes caused by alcohol, alcoholic myopathy, and alcoholic gastritis), and obesity. We used Charlson Comorbidity Index (CCI) scores as a measure of the burden of comorbidity. The CCI scoring system allocates from one to six points to a range of diseases, as components of a summed aggregate score. Patients were categorized into three subgroups according to their calculated CCI score: low (no comorbidities) = CCI score of 0; medium = CCI score of 1–2; or high = CCI score of 3 or more (Supplementary Table S8).

### Statistics

We calculated prevalence proportions (PPs) and prevalence ratios (PRs) comparing characteristics of PCCRCs vs. dCRCs among T2D patients. The robust Poisson method was used to calculate PRs and associated 95% confidence intervals (CIs) [20].

We also followed the cohort of T2D patients from the date of CRC diagnosis until first occurrence of all-cause death, emigration, or the administrative end of follow-up (December 31, 2015). Survival probabilities were computed using the Kaplan-Meier technique. We used Cox-proportional hazard regression models to compute hazard ratios (HRs). No violation to the proportional hazard assumption was found. Crude and adjusted HRs and associated 95% CIs were used as an estimate of the mortality rate ratio (MRR) comparing PCCRC in T2D patients with dCRC in T2D patients. We constructed two separate regression models adjusted for potential confounders of the association between PCCRC and death. The first model included age, sex, and year of CRC diagnosis. To include the effect of more advanced CRC stage in PCCRC vs. dCRC, as suggested in a previous study [21], we constructed a second model including age, sex, year of CRC diagnosis, and CRC stage at diagnosis. Survival probabilities and HRs were computed for one year and for one to five years after a CRC diagnosis. In addition, we investigated 90-day and five-year survival after a PCCRC/dCRC diagnosis.

### Sensitivity analysis

Due to changing data availability over the study period, we identified T2D patients using both ICD-codes in the DNPR during 1977–2015 and prescription redemptions recorded in the DNHSPD during 2004–2015. We evaluated the impact of our identification method by conducting a sensitivity analysis restricted to patients who underwent colonoscopies during 2005–2015. In this analysis, we further evaluated the impact of our diabetes categorization (types 1 and 2) by extending the definition of type 1 diabetes mellitus to include patients with a DNPR diagnosis of diabetes before age 30, using insulin monotherapy, and with no history of oral glucose-lowering medications before the date of CRC diagnosis.

All data management and statistical analyses were conducted using Stata Version 15 (StataCorp, College Station, Texas, USA).

## Results

### Characteristics of PCCRCs *vs.* detected colorectal cancers

We identified 3088 T2D patients diagnosed with CRC during 1995–2015, who underwent a colonoscopy before their diagnosis. Characteristics of all T2D patients included in the study are shown in Table 1. A total of 250 (8.1%) patients were categorized as having PCCRC, and 2.838 (91.9%) patients were categorized as having dCRC. Although we observed a predominance of men in the study cohort (61%), PCCRC patients were more likely to be female than dCRC patients. Median age at CRC diagnosis was 74 years for patients with PCCRC and 73 years for patients with dCRC. The majority of PCCRCs (55%) were diagnosed during 2011-2015; however, the prevalence of PCCRC was higher than the prevalence of dCRCs at the beginning of the study period in the 1990s. Thus, PRs comparing PCCRC *vs.* dCRC declined over time, from 3.68 (95% CI 1.94–6.97) in 1995–2000 to 0.97 (95% CI 0.86–1.09) in 2011–2015 (Table 1).

**Table 1:** Characteristics of patients with Type two diabetes mellitus (T2D) and colorectal cancer (CRC) categorized as post-colonoscopy CRC (PCCRC) or detected CRC (dCRC)

	PCCRC, *n* (%)	dCRC, *n* (%)	PR (95% CI)^a^
Total	250 (8.1)	2838 (91.9)	
Male	137 (54.8)	1756 (61.9)	0.89 (0.79–0.99)
Female	113 (45.2)	1082 (38.1)	1.19 (1.03–1.37)
Age at CRC diagnosis, years			
Median age at diagnosis (IQR^b^)	73.9 (68.6–79.6)	72.5 (66.5–78.8)	
0–59	15 (6.0)	248 (8.8)	0.69 (0.41–1.14)
60–69	63 (25.2)	796 (28.0)	0.90 (0.72–1.12)
70 +	172 (68.8)	1794 (63.2)	1.09 (1.00–1.19)
Year of CRC diagnosis			
1995–2000	12 (4.8)	37 (1.3)	3.68 (1.94–6.97)
2000–2005	30 (12.0)	323 (11.4)	1.05 (0.74–1.50)
2006–2010	70 (28.0)	863 (30.4)	0.92 (0.75–1.13)
2011–2015	138 (55.2)	1615 (56.9)	0.97 (0.86–1.09)
T2D identification^c^			
ICD code	108 (43.2)	958 (33.8)	1.30 (1.10–1.49)
Prescription	35 (14.0)	342 (12.0)	1.16 (0.84–1.60)
Both	107 (42.8)	1538 (54.2)	0.79 (0.68–0.92)
T2D duration^d^, years			
< 1	12 (4.8)	549 (19.3)	0.24 (0.14–0.43)
1–5	87 (34.8)	911 (32.1)	1.08 (0.91–1.29)
6–10	79 (31.6)	737 (26.0)	1.21 (1.00–1.47)
> 10	72 (28.8)	641 (22.6)	1.27 (1.04–1.56)
Selected comorbidities^e^			
Chronic obstructive pulmonary disease	31 (12.4)	227 (8.0)	1.55 (1.09–2.21)
Atrial fibrillation/flutter	48 (19.2)	406 (14.3)	1.34 (1.02–1.76)
Cardiovascular diseases	162 (64.8)	1570 (55.3)	1.17 (1.06–1.30)
Renal disease	21 (8.4)	149 (5.3)	1.60 (1.03–2.48)
Alcohol-related diseases	11 (4.4)	127 (4.5)	0.98 (0.54–1.80)
Obesity	34 (13.6)	369 (13.0)	1.04 (0.75–1.45)
CCI score^f^			
Low	74 (29.6)	1175 (41.4)	0.71 (0.59–0.87)
Medium	102 (40.8)	1129 (39.8)	1.03 (0.88–1.20)
High	74 (29.6)	534 (18.8)	1.57 (1.28–1.93)
CRC stage at diagnosis			
Localized	86 (34.4)	1123 (39.6)	0.87 (0.73–1.04)
Regional	35 (14.0)	545 (19.2)	0.73 (0.53–1.00)
Metastatic	54 (21.6)	597 (21.0)	1.03 (0.80–1.31)
Unknown	75 (30.0)	573 (20.2)	1.49 (1.21–1.82)
CRC site			
Proximal colon	136 (54.4)	1078 (40.0)	1.43 (1.27–1.62)
Distal colon	49 (19.6)	901 (31.7)	0.62 (0.48–0.80)
Rectum	40 (16)	729 (25.7)	0.62 (0.47–0.83)
Unspecified or more than one site	25 (10)	130 (4.6)	2.18 (1.45–3.28)
CRC histology			
Adenocarcinoma	195 (78)	2365 (83.3)	0.94 (0.88–1.00)
Polyp adenocarcinoma	10 (4)	177 (6.2)	0.64 (0.34–1.20)
Mucinous carcinoma	7 (2.8)	108 (3.8)	0.74 (0.35–1.56)
Signet ring	N/A	10 (0.4)	1.14 (0.15–8.83)
Neuroendocrine	N/A	18 (0.6)	0.63 (0.08–4.71)
Other histology^g^	22 (8.8)	74 (2.6)	3.37 (2.13–5.34)
Not histologically verified	14 (5.6)	86 (3.0)	1.85 (1.07–3.20)
CRC Surgery			
Colorectal surgeries^h^	221 (88.4)	2520 (88.8)	1.00 (0.95–1.04)
No surgery	29 (11.6)	318 (11.2)	1.04 (0.72–1.48)
Mismatch repair status^i^			
MMR proficient	64 (53.8)	996 (55.6)	1.00 (0.81–1.15)
MMR deficient	55 (46.2)	794 (44.4)	1.04 (0.85–1.27)
Histologically verified polyps^j^			
Yes	158 (63.2)	819 (28.9)	2.19 (1.96–2.45)
No	92 (36.8)	2019 (71.1)	0.52 (0.44–0.61)

Prevalence ratios (PRs) and associated 95% confidence intervals (CIs) compare T2D patients with PCCRC to T2D patients with dCRC. Denmark, 1995–2015. Numbers < 5 are marked “N/A” to ensure anonymity. CRCs diagnosed 7–36 months after a negative colonoscopy. CRCs diagnosed within 6 months after a colonoscopy

^a^PRs and 95% CIs from robust Poisson regression testing associations of individual characteristics of T2D patients with PCCRC compared to those of T2D patients with dCRC

^b^IQR: interquartile range

^c^Recorded before or within 90 days after a first colonoscopy

^d^Time from initial T2D identification until CRC diagnosis

^e^Recorded before the date of PCCRC/dCRC diagnosis

^f^Charlson Comorbidity Index score: low = CCI score of 0; medium = CCI score of 1–2; high = CCI score of 3 or more

^g^Including sarcomas, lymphomas, metastases, and unspecified histology

^h^Including total colectomy, partial colectomy, rectal resection, and other colorectal surgeries

^i^Restricted to CRCs with an available test for mismatch repair (MMR) status (*n* = 61.8% of included patients). CRCs demonstrating absent nuclear expression of one or more MMR proteins (MLH1, MSH2, MSH6, and PMS2) were considered MMR-deficient

^j^Histologically verified colorectal polyps (conventional adenomas and serrated polyps) or polypectomies recorded before the date of CRC diagnosis

Increased duration of T2D seemed to be associated with increasing prevalence of PCCRC. PCCRC patients were more likely than dCRC patients to have a high CCI score (the complete distribution of comorbidities included in the CCI is displayed in Supplementary Table 8), although atrial fibrillation/flutter and other cardiovascular diseases were highly prevalent in the entire T2D cohort. No difference was observed for diagnoses of obesity. In addition, we observed similar distributions of stage, histology, surgery, and MMR status among PCCRCs and dCRCs (Table 1). However, an increased prevalence of proximally located CRCs was observed for PCCRCs (PR: 1.43 [95% CI 1.27–1.62]). Similarly, PCCRC patients had an increased prevalence of histologically verified colorectal polyps and polypectomies recorded before the date of their CRC diagnosis (PR 2.19 [95% CI 1.96–2.45]) (Table 1).

### Survival after post-colonoscopy *vs.* detected colorectal cancers

One-year survival after a CRC diagnosis was lower among T2D patients with PCCRC than among T2D patients with dCRC (Table 2). The crude HR for death at one year, comparing PCCRC with dCRC patients, was 1.85 (95% CI 1.47–2.31). When adjusting for age, sex, and year of CRC diagnosis, the association remained (HR 1.77 [95% CI 1.42–2.23]). The association also remained after inclusion of CRC stage in the regression model (HR 1.69 [95% CI 1.35–2.12]) (Table 2). Our additional analysis of 90-day survival after a CRC diagnosis showed the same pattern, with a crude HR of 1.95 (95% CI 1.43–2.65) and an age-, sex-, year-of-CRC-diagnosis-, and stage-adjusted HR of 1.71 (95% CI 1.25–2.33) (Supplementary Table 1).

**Table 2:** Number of deaths, survival probabilities, and hazard ratios (HRs) as a measure of mortality rate ratios in patients with diabetes mellitus type 2 (T2D) and colorectal cancer (CRC) categorized as post-colonoscopy CRC (PCCRC) or detected CRC (dCRC), Denmark 1995–2015

First year after CRC diagnosis							
	PCCRC		dCRC				
	No. of deaths	1 year survival, % (95% CI)	No. of deaths	1 year survival, % (95% CI)	Crude HR (95% CI)	Adjusted HR^a^ (95% CI)	Adjusted HR^b^ (95% CI)
Total	87	63 (56–69)	603	78 (76–79)	1.85 (1.47–2.31)	1.77 (1.42–2.23)	1.69 (1.35–2.12)
Male	47	64 (55–71)	352	79 (77–81)	1.91 (1.41–2.59)	1.82 (1.34–2.47)	1.78 (1.31–2.42)
Female	40	62 (52–70)	251	76 (73–78)	1.73 (1.24–2.42)	1.73 (1.24–2.42)	1.63 (1.16–2.28)
Age at CRC diagnosis, y							
0–59	N/A	85 (51–96)	39	83 (78–87)	0.90 (0.22–3.74)	0.89 (0.21–3.73)	0.97 (2.23–4.03)
60–69	N/A	70 (56–80)	118	84 (82–87)	2.12 (1.29–3.48)	1.98 (1.19–3.30)	1.78 (1.07–2.98)
70 +	67	59 (51–66)	446	74 (72–76)	1.78 (1.38–2.31)	1.77 (1.37–2.30)	1.71 (1.32–2.21)
Year of CRC diagnosis							
1995–2000	N/A	67 (34–86)	10	73 (56–84)	1.19 (0.37–3.81)	1.21 (0.37–3.99)	1.36 (0.37–4.94)
2000–2005	N/A	77 (57–88)	98	70 (64–74)	0.71 (0.33–1.53)	0.69 (0.32–1.49)	0.63 (0.29–1.37)
2006–2010	29	57 (45–68)	208	76 (73–79)	2.05 (1.39–3.02)	2.01 (1.36–2.96)	1.84 (1.25–2.72)
2011–2015	47	62 (53–70)	287	80 (78–82)	2.22 (1.63–3.03)	2.12 (1.55–2.88)	2.06 (1.51–2.81)
T2D identification							
ICD code	37	64 (54–73)	255	73 (70–75)	1.33 (0.95–1.88)	1.27 (0.90–1.80)	1.21 (0.85–1.71)
Prescription	8	74 (54–86)	63	81 (76–85)	1.45 (0.70–3.03)	1.53 (0.73–3.19)	1.36 (0.64–2.89)
Both	42	58 (48–67)	285	80 (78–82)	2.56 (1.85–3.54)	2.58 (1.86–3.57)	2.36 (1.70–3.07)
T2D duration, y							
< 1	6	46 (17–71)	123	77 (72–80)	2.66 (1.17–6.04)	2.24 (0.95–5.27)	1.84 (0.75–4.49)
1–5	28	66 (55–75)	185	79 (75–81)	1.70 (1.14–2.53)	1.73 (1.16–2.58)	1.80 (1.20–2.70)
6–10	27	62 (50–72)	155	78 (74–80)	1.97 (1.31–2.96)	1.96 (1.30–2.97)	1.75 (1.16–2.66)
> 10	26	61 (49–72)	140	77 (73–79)	1.80 (1.18–2.73)	1.60 (1.04–2.45)	1.50 (0.98–2.29)
CCI score							
Low	11	60 (40–76)	82	83 (80–87)	2.95 (1.57–5.53)	3.64 (1.89–7.01)	3.27 (1.68–6.36)
Medium	25	65 (54–75)	222	77 (74–80)	1.65 (1.09–2.50)	1.58 (1.04–2.40)	1.53 (1.00–2.34)
High	51	62 (53–70)	299	75 (72–77)	1.69 (1.25–2.27)	1.63 (1.21–2.20)	1.49 (1.10–2.01)

Numbers < 5 marked with “N/A” to ensure anonymity. Colorectal cancer diagnosed 7–36 months after a negative colonoscopy. Colorectal cancer diagnosed within 6 months after a preceding colonoscopy

^a^Adjusted for age, sex, and year of CRC diagnosis

^b^Adjusted for age, sex, year of CRC diagnosis, and CRC stage

^c^Charlson Comorbidity Index score: low = CCI score of 0; medium = CCI score of 1–2; high = CCI score of 3 or more

Abbreviations: Hazard ratios (HRs); Charlson Comorbidity Index (CCI); colorectal cancer (CRC); post-colonoscopy colorectal cancer (PCCRC); detected colorectal cancer (dCRC); confidence intervals (CIs)

One to five years after a CRC diagnosis, survival remained lower among T2D patients with PCCRC than among T2D patients with dCRC (Table 3). The estimated one- to five-year survival probability was 44% (95% CI 34–53) for patients with PCCRC and 54% (95% CI 52–57) for patients with dCRCs (Table 3). The corresponding adjusted HRs were 1.46 (95% CI 1.12–1.89) after adjusting for age, sex, and year of CRC, and 1.55 (95% CI 1.19–2.01) after adding stage to the model (Table 3). In our additional analysis of five-year survival, survival probabilities remained lower in the group of PCCRC patients. The corresponding five-year HR adjusted for age, sex, year of CRC diagnosis, and stage was 1.66 (96% CI 1.40–1.97) (Supplementary Table 2).

**Table 3:** Number of deaths, survival probabilities, and hazard ratios (HRs) as a measure of mortality rate ratios in patients with diabetes mellitus type 2 (T2D) and colorectal cancer (CRC) categorized as post-colonoscopy CRC (PCCRC) or detected CRC (dCRC), Denmark 1995–2015

One to five years after CRC diagnosis							
	PCCRC		dCRC				
	No. of deaths	1–5 year survival, % (95% CI)	No. of deaths	1–5 year survival, % (95% CI)	Crude HR (95% CI)	Adjusted HR^a^ (95% CI)	Adjusted HR^b^ (95% CI)
Total	63	44 (34–53)	672	54 (52–57)	1.44 (1.11–1.87)	1.46 (1.12–1.89)	1.55 (1.19–2.01)
Male	44	35 (24–47)	449	51 (48–54)	1.72 (1.26–2.34)	1.75 (1.28–2.40)	1.80 (1.31–2.46)
Female	19	57 (40–71)	223	60 (55–64)	1.11 (0.70–1.77)	1.06 (0.67–1.70)	1.17 (0.73–1.89)
Age at CRC diagnosis, y							
0–59	N/A	76 (33–94)	55	61 (53–69)	0.53 (0.13–2.18)	0.54 (1.13–2.30)	0.58 (0.14–2.41)
60–69	N/A	46 (27–63)	201	55 (51–60)	1.45 (0.88–2.38)	1.48 (0.90–2.46)	1.60 (0.97–2.65)
70 +	44	39 (27–51)	416	52 (49–56)	1.56 (1.14–2.12)	1.58 (1.15–2.14)	1.62 (1.18–2.22)
Year of CRC diagnosis							
1995–2000	N/A	63 (23–86)	14	48 (29–65)	0.71 (0.20–2.47)	0.75 (0.21–2.74)	1.12 (0.29–4.35)
2000–2005	N/A	30 (14–49)	83	63 (56–69)	2.50 (1.46–4.28)	2.51 (1.46–4.31)	2.56 (1.48–4.42)
2006–2010	19	51 (35–66)	255	61 (57–65)	1.48 (0.93–2.36)	1.52 (0.95–2.42)	1.59 (1.00–2.55)
2011–2015	25	34 (16–54)	320	28 (21–36)	1.17 (0.78–1.76)	1.21 (0.80–1.82)	1.27 (0.84–1.90)
T2D identification							
ICD code	35	39 (27–52)	243	57 (52–61)	1.76 (1.23–2.50)	1.76 (1.22–2.54)	2.04 (1.41–2.95)
Prescription	5	67 (36–85)	75	56 (48–64)	0.84 (0.34–2.08)	0.76 (0.30–1.89)	0.73 (0.29–1.82)
Both	23	44 (27–59)	354	52 (48–56)	1.36 (0.89–2.08)	1.42 (0.93–2.17)	1.43 (0.93–2.19)
T2D duration, y							
< 1	N/A	9 (1–33)	138	45 (40–50)	3.92 (1.45–10.60)	6.96 (2.30–21.05)	6.06 (2.08–17.68)
1–5	25	30 (20–41)	193	48 (44–52)	1.15 (1.01–2.32)	1.56 (1.02–2.37)	1.78 (1.17–2.73)
6–10	18	28 (17–41)	192	35 (31–40)	1.17 (0.72–1.90)	1.22 (0.75–1.98)	1.15 (0.76–1.87)
> 10	N/A	29 (17–42)	149	37 (32–42)	1.27 (0.76–2.13)	1.28 (0.75–2.20)	1.33 (0.78–2.28)
CCI score							
Low	N/A	41 (22–60)	97	55 (50–60)	1.23 (0.45–3.36)	1.09 (0.38–3.12)	1.13 (0.40–3.18)
Medium	N/A	32 (21–45)	237	44 (40–47)	1.18 (0.73–1.92)	1.16 (0.71–1.86)	1.38 (0.85–2.24)
High	41	23 (15–31)	338	35 (32–39)	1.48 (1.07–2.05)	1.55 (1.11–2.54)	1.54 (1.11–2.13)

Numbers < 5 marked with “N/A” to ensure anonymity. Colorectal cancer diagnosed 7–36 months after a negative colonoscopy. Colorectal cancer diagnosed within 6 months after a preceding colonoscopy

^a^Adjusted for age, sex, and year of CRC diagnosis

^b^Adjusted for age, sex, year of CRC diagnosis, and CRC stage

^c^Charlson Comorbidity Index score: low = CCI score of 0; medium = CCI score of 1–2; high = CCI score of 3 or more

*HRs* Hazard ratios; *CCI* Charlson Comorbidity Index; *CRC* colorectal cancer; *PCCRC* post-colonoscopy colorectal cancer; *dCRC* detected colorectal cancer; *CIs* confidence intervals

PCCRC patients had a poorer survival than dCRC patients across all strata of CCI scores (Tables 2 and 3).

### Sensitivity analysis

The sensitivity analysis revealed no material differences from our main analysis. The results are shown in Supplementary Tables 3, 4, 5.

## Discussion

In this registry-based nationwide cohort study of 3088 T2D patients diagnosed with either PCCRC or dCRC, we found that patients with PCCRC had an elevated prevalence of proximally located CRCs. PCCRCs and dCRCs did not differ in terms of stage, histology, and MMR status. The proportion of PCCRCs compared with dCRCs decreased over time. In all time-intervals analyzed, we found poorer survival in the PCCRC group compared with the dCRC group.

Previous research has suggested incidence of CRC and colorectal polyps to be linked with prevalent T2D as well as obesity [22–24]. Furthermore, Kort et al. studied the endoscopic phenotype and histopathology of colorectal polyps in patients with and without diabetes mellitus (including 3654 patients who underwent colonoscopy) [11]. They observed that diabetic patients more often had multiple and proximally located adenomas compared to non-diabetic patients. Our study found T2D-related PCCRCs to be more frequently diagnosed after resection of colorectal polyps than dCRCs. Considering the findings by Kort et al*.*, our results study may reflect a predominance of T2D-related PCCRC caused by missed lesions at the index colonoscopy and particularly those missed in the proximal colon. The results of the present study thus expand those of Kort et al*.* and indicate the importance of specific surveillance and thorough preparation of patients with diabetes mellitus for colonoscopies.

We found that the proportion of PCCRCs decreased over time. This may indicate improved quality of colonoscopies [25]. Since CRC screening was introduced in Denmark in March 2014, there has been an increased focus on improving colonoscopy quality. In a large cohort study conducted using data from 2001 to 2015, Pedersen *et al.* found that the 3-year rate of PCCRC was generally higher in Denmark than in England, though declining over time [26]. Troelsen et al. reported the same decreasing trend [8].

Our survival analyses indicated that PCCRC was associated with poorer one-year and 1-to 5-year survival than dCRC. Despite conflicting results, the majority of previous studies found no difference in survival for patients with dCRC and PCCRC [2, 27–32]. These studies have led to the interpretation of PCCRC as CRC arising mainly from missed or insufficiently resected precursor lesions. In contrast, our findings indicate that T2D patients with PCCRC have a poorer prognosis than those with dCRC. This could indicate altered biology in the T2D patients related to hyperinsulinemia, hyperglycaemia, and changes in microbiota. Some studies suggested that long-term T2D might interfere with the molecular pathways of CRC, and that insulin sensitivity in T2D patients may lead to chronic compensatory hyperinsulinemia. Concurrent circulating levels of the insulin-like growth factor-1 receptor are associated with colorectal cancer development [33, 34]. Still, we found no difference between PCCRCs and dCRCs according to MMR status which has been associated with rapid tumor growth in previous research [35–37]. Our results concerning T2D-related CRC biology are thus contradictory and future studies on molecular characteristics are needed.

Study strengths include a population-based design and setting in Denmark, which has a well-organized health system generating continuously updated and high-quality data. Our study also has several limitations. First, we lacked data on quality of bowel preparation, completeness of the examination, endoscopist-specific adenoma detection rates, and information on surveillance regimens. This information is crucial for determining the clinical development of PCCRC, mainly because of possibly impaired bowel preparation prior to colonoscopy among T2D patients. Second, data availability differed during the study period, which may have affected our results. Data on prescriptions for diabetic medications were not available before 2004, when the DNHSPD was established [38].Thus up to 2004, our cohort only consisted of T2D patients with a diabetes diagnosis registered in the DNPR. For this reason, our study may have missed a proportion of T2D patients treated by general practitioners during 1995–2003. However, a sensitivity analysis restricted to colonoscopies performed during 2005–2012 indicated that this shortcoming had only a minor impact on our results.

In conclusion, our findings suggest overlooked colorectal lesions as a predominant explanation for T2D-related PCCRC, although altered tumor progression cannot be ruled out. This points to the need for increased awareness of colonoscopy quality and perhaps shorter intervals between colonoscopies among patients with T2D. Future research concerning T2D-related PCCRC should include information on quality of bowel preparation, completeness of colonoscopies, endoscopist-specific adenoma detection rates, and information on surveillance regimens.

### Supplementary Information

Below is the link to the electronic supplementary material.

Supplementary file1 (DOCX 87 KB)

## Data Availability

Data are available to legal residents of Denmark; however, only by application to the Danish Health Data Authority (https://sundhedsdatastyrelsen.dk/da/forskerservice/ansog-om-data) and the contributing hospitals. Hence, the data cannot be acquired directly by the authors.

## Abbreviations

CCI: Charlson Comorbidity Index
CRC: Colorectal cancer
CI: Confidence interval
T2D: Type 2 diabetes mellitus
dCRC: Detected colorectal cancer
HR: Hazard ratio
MMR: Mismatch repair
PCCRC: Post-colonoscopy colorectal cancer
PP: Prevalence proportion
PR: Prevalence ratio
SNOMED: Systematized Nomenclature of Medicine
DCR: Danish Cancer Registry
CRS: Danish Civil Registration System
ICD: International Classification of Diseases
ICD-8: Eighth Revision
ICD-10: Tenth Revision
DNHSPD: Danish National Health Service Prescription Database
DPR: Danish National Pathology Registry
DNPR: Danish National Patient Registry
WEO: World Endoscopy Organization
