# Supplementary material for: Type 2 diabetes mellitus and post-colonoscopy colorectal cancer: clinical and molecular characteristics and survival
Source: Cancer Causes Control. 2024 Mar 14;35(7):1043–52. doi: 10.1007/s10552-024-01861-9 (PMC11217032; doi:10.1007/s10552-024-01861-9)
Supplement: Supplementary file 1 — Supplementary file1 (DOCX 87 KB) [file 10552_2024_1861_MOESM1_ESM.docx]

# Supplementary Tables

| Supplementary Table 1**.** Number of deaths, survival probabilities, and hazard ratios (HRs) as a measure of mortality rate ratios in patients with diabetes mellitus type 2 (T2D) and colorectal cancer (CRC) categorized as post-colonoscopy CRC (PCCRC)^1^ or detected CRC (dCRC)^2^, Denmark, 1995-2015. Numbers <5 marked with “N/A” to ensure anonymity. | | | | | | | | | | | |
| --- | --- | --- | --- | --- | --- | --- | --- | --- | --- | --- | --- |
| **90 days after CRC diagnosis** | | | | | | | | | | | |
|  | **PCCRC** | |  | **dCRC** | |  |  |  |  |  |  |
|  | **No. of deaths** | **90 days survival, % (95% CI)** |  | **No. of deaths** | **90 days survival, % (95% CI)** |  | **Crude HR**  **(95 % CI)** |  | **Adjusted HR^3^**  **(95% CI)** |  | **Adjusted HR^4^**  **(95% CI)** |
| **Total**  Male  Female | **47**  25  22 | **81 (75-85)**  82 (74-87)  80 (71-86) |  | **295**  168  127 | **89 (88-91)**  90 (89-92)  88 (86-90) |  | **1.95 (1.43-2.65)**  2.03 (1.33-3.08)  1.82 (1.16-2.86) |  | **1.87 (1.37-2.54)**  1.87 (1.22-2.86)  1.86 (1.18-2.93) |  | **1.71 (1.25-2.33)**  1.76 (1.15-2.69)  1.72 (1.09-2.72) |
| **Age at CRC diagnosis, years**  0-59  60-69  70+ | N/A  N/A  37 | 93 (61-99)  86 (74-92)  78 (71-83) |  | 15  57  223 | 94 (90-96)  93 (91-94)  87 (86-89) |  | 1.14 (0.15-8.62)  2.12 (1.05-4.28)  1.88 (1.33-2.66) |  | 1.02 (0.13-8.10)  1.91 (0.93-3.95)  1.88 (1.33-2.67) |  | 1.33 (0.17-10.19)  1.73 (0.84-3.58)  1.72 (1.21-2.44) |
| **Year of CRC diagnosis**  1995-2000  2000-2005  2006-2010  2011-2015 | N/A  N/A  17  27 | 92 (54-99)  93 (76-98)  75 (63-84)  80 (72-86) |  | 7  48  115  125 | 81 (64-91)  85 (81-89)  87 (84-89)  92 (91-93) |  | 0.44 (0.05-3.58)  0.42 (0.10-1.74)  2.05 (1.23-3.41)  2.77 (1.83-4.20) |  | 0.35 (0.04-3.05)  0.41 (0.10-1.67)  1.99 (1.20-3.31)  2.59 (1.70-3.93) |  | 0.58 (0.05-7.04)  0.38 (0.09-1.59)  1.72 (1.03-2.88)  2.40 (1.58-3.66) |
| **T2D identification**  ICD code  Prescription  Both | 16  5  26 | 85 (77-90)  85 (66-93)  75 (65-82) |  | 139  31  125 | 85 (83-87)  91 (87-93)  92 (90-93) |  | 1.04 (0.62-1.75)  1.64 (0.64-4.22)  3.43 (2.25-5.24) |  | 1.00 (0.59-1.68)  1.78 (0.69-4.60)  3.30 (2.16-5.05) |  | 0.93 (0.55-1.56)  1.45 (0.56-3.78)  2.89 (1.88-4.44) |
| **T2D**  **duration, years**  <1  1-5  6-10  >10 | N/A  16  16  N/A | 83 (48-96)  81 (71-88)  79 (68-87)  81 (70-89) |  | 60  94  73  68 | 89 (86-91)  90 (87-91)  90 (87-92)  89 (86-91) |  | 1.70 (0.42-6.96)  1.83 (1.08-3.12)  2.31 (1.35-3.97)  1.82 (1.01-3.30) |  | 1.31 (0.30-5.71)  1.83 (1.07-3.12)  2.21 (1.28-3.84)  1.67 (0.91-3.05) |  | 1.04 (0.22-4.87)  1.76 (1.03-3.01)  1.95 (1.12-3.38)  1.56 (0.85-2.86) |
| **Supplementary Table 1, continued** | | | | | | | | | | | |
| ^1^Colorectal cancer diagnosed 7-36 months after a negative colonoscopy.  ^2^Colorectal cancer diagnosed within 6 months after a preceding colonoscopy.  ^3^Adjusted for age, sex, and year of CRC diagnosis.  ^4^Adjusted for age, sex, year of CRC diagnosis, and CRC stage.  Abbreviations: Hazard ratios (HRs); colorectal cancer (CRC); post-colonoscopy colorectal cancer (PCCRC); detected colorectal cancer (dCRC); confidence intervals (CIs) | | | | | | | | | | | |

| Supplementary Table 2**.** Number of deaths, survival probabilities, and hazard ratios (HRs) as a measure of mortality rate ratios in patients with diabetes mellitus type 2 (T2D) and colorectal cancer (CRC) categorized as post-colonoscopy CRC (PCCRC)^1^ or detected CRC (dCRC)^2^, Denmark, 1995-2015. Numbers <5 marked with “N/A” to ensure anonymity. | | | | | | | | | | | |
| --- | --- | --- | --- | --- | --- | --- | --- | --- | --- | --- | --- |
| **Five years after CRC diagnosis** | | | | | | | | | | | |
|  | **PCCRC** | |  | **dCRC** | |  |  |  |  |  |  |
|  | **No. of deaths** | **5-year survival, % (95% CI)** |  | **No. of deaths** | **5-year survival, % (95% CI)** |  | **Crude HR**  **(95 % CI)** |  | **Adjusted HR^3^ (95% CI)** |  | **Adjusted HR^4^ (95% CI)** |
| **Total**  Male  Female | **150**  91  59 | **28 (21-35)**  22 (15-31)  35 (24-46) |  | **1275**  801  474 | **42 (40-44)**  40 (37-43)  45 (42-49) |  | **1.65 (1.39-1.96)**  1.81 (1.46-2.25)  1.47 (1.12-1.92) |  | **1.63 (1.38- 1.94)**  1.79 (1.44-2.23)  1.45 (1.10-1.90) |  | **1.66 (1.40-1.97)**  1.80 (1.45-2.25)  1.49 (1.13-1.95) |
| **Age at CRC diagnosis, years**  0-59  60-69  70+ | N/A  N/A  111 | 65 (30-85)  32 (19-46)  23 (16-31) |  | 94  319  862 | 51 (43-58)  46 (42-51)  38 (36-41) |  | 0.67 (0.25-1.82)  1.73 (1.22-2.45)  1.68 (1.38-2.05) |  | 0.67 (0.24-1.87)  1.74 (1.22-2.49)  1.69 (1.39-2.06) |  | 0.75 (0.27-2.05)  1.80 (1.26-2.57)  1.71 (1.40-2.09) |
| **Year of CRC diagnosis**  1995-2000  2000-2005  2006-2010  2011-2015 | 7  23  48  72 | 42 (15-67)  23 (10-39)  29 (19-40)  21 (10-35) |  | 24  181  463  607 | 35 (20-50)  44 (38-49)  46 (43-50)  23 (17-29) |  | 0.92 (0.40-2.14)  1.43 (0.93-2.21)  1.78 (1.32-2.39)  1.69 (1.33-2.16) |  | 0.95 (0.40-2.27)  1.41 (0.91-2.18)  1.78 (1.32-2.40)  1.68 (1.31-2.14) |  | 1.14 (0.47-2.77)  1.37 (0.88-2.12)  1.75 (1.30-2.36)  1.73 (1.35-2.21) |
| **T2D identification**  ICD code  Prescription  Both | 72  13  65 | 25 (17-35)  49 (27-68)  25 (16-36) |  | 498  138  639 | 41 (38-45)  45 (39-52)  41 (38-45) |  | 1.51 (1.18-1.94)  1.13 (0.64-2.00)  1.95 (1.51-2.52) |  | 1.49 (1.15-1.91)  1.15 (0.65-2.04)  1.97 (1.53-2.55) |  | 1.55 (1.20-2.00)  1.09 (0.61-1.94)  1.90 (1.47-2.46) |
| **T2D**  **duration, years**  <1  1-5  6-10  >10 | 10  53  45  42 | 9 (5-33)  30 (19-41)  28 (16-40)  29 (17-42) |  | 261  378  347  289 | 45 (40-49)  47 (43-51)  35 (31-39)  37 (32-41) |  | 3.06 (1.62-5.77)  1.61 (1.21-2.15)  1.55 (1.13-2.11)  1.55 (1.12-2.14) |  | 3.26 (1.67-6.34)  1.63 (1.22-2.18)  1.56 (1.14-2.14)  1.47 (1.06-2.06) |  | 2.93 (1.51-5.70)  1.79 (1.33-2.39)  1.45 (1.06-1.98)  1.47 (1.06-2.05) |
| **Supplementary Table 2, continued** | | | | | | | | | | | |
| ^1^Colorectal cancer diagnosed 7-36 months after a negative colonoscopy.  ^2^Colorectal cancer diagnosed within 6 months after a preceding colonoscopy.  ^3^Adjusted for age, sex, and year of CRC diagnosis.  ^4^Adjusted for age, sex, year of CRC diagnosis, and CRC stage.  Abbreviations: Hazard ratios (HRs); colorectal cancer (CRC); post-colonoscopy colorectal cancer (PCCRC); detected colorectal cancer (dCRC); confidence intervals (CIs) | | | | | | | | | | | |

| Supplementary Table 3**.** Characteristics of patients with diabetes mellitus Type 2 (T2D) and colorectal cancer (CRC) categorized as post-colonoscopy CRC (PCCRC)^1^ or detected CRC (dCRC)^2^. Prevalence ratios (PR) and associated 95% confidence interval (CI) comparing T2D patients with PCCRC to T2D patients with dCRC, Denmark, 2005-2015. Numbers <5 marked with “N/A” to ensure anonymity. | | | | | | |
| --- | --- | --- | --- | --- | --- | --- |
|  |  | **PCCRC, n (%)** |  | **dCRC, n (%)** |  | **PR (95% CI)^3^** |
| **Total**  Male  Female |  | **215 (7.7)**  113 (52.6)  102 (47.4) |  | **2,583 (92.3)**  1,604 (62.1)  979 (37.9) |  | 0.85 (0.74-0.96)  1.25 (1.08-1.45) |
| **Age at CRC diagnosis, years**  Median age at diagnosis (IQR^4^)  0-59  60-69  70+ |  | 74.2 (68.6-80.2)  13 (6.1)  50 (23.3)  152 (70.7) |  | 72.5 (66.6-78.8)  209 (8.9)  735 (28.5)  1,639 (63.5) |  | 0.74 (0.43-1.29)  0.82 (0.64-1.05)  1.11 (1.02-1.22) |
| **Year of CRC diagnosis**  2005  2006-2010  2011-2015 |  | 7 (3.3)  70 (32.6)  138 (64.2) |  | 105 (4.1)  863 (33.4)  1615 (62.5) |  | 0.80 (0.38-1.70)  0.97 (0.78-1.19)  1.03 (0.92-1.14) |
| **T2D identification^5^**  ICD code  Prescription  Both |  | 73 (34.0)  35 (16.3)  107 (49.8) |  | 755 (29.2)  331 (12.8)  1,497 (58.0) |  | 1.16 (0.95-1.41)  1.27 (0.92–1.75)  0.86 (0.75-0.99) |
| **T2D duration^6^, years**  <1  1-5  6-10  >10 |  | 6 (2.8)  69 (32.1)  78 (36.3)  62 (28.8) |  | 460 (17.8)  833 (32.3)  695 (26.9)  595 (23.0) |  | 0.16 (0.07-0.35)  1.00 (0.81-1.22)  1.35 (1.12-1.63)  1.25 (1.00-1.56) |
| **Selected Comorbidities^7^**  Chronic obstructive pulmonary disease  Atrial fibrillation/flutter  Cardiovascular diseases  Renal disease  Alcohol-related diseases |  | 26 (12.9)  44 (20.5)  142 (66.1)  17 (7.9)  8 (3.7) |  | 212 (8.2)  377 (14.6)  1,452 (56.2)  144 (5.6)  119 (4.6) |  | 1.47 (1.00-2.16)  1.40 (1.06-1.85)  1.17 (1.06-1.30)  1.42 (0.87-2.30)  0.81 (0.40-1.63) |
| **CCI score^8^**  Low  Medium  High |  | 65 (30.2)  86 (40.0)  64 (29.8) |  | 1.078 (41.7)  1.020 (39.5)  485 (18.8) |  | 0.72 (0.59-0.89)  1.01 (0.85-1.20)  1.59 (1.27-1.98) |
| **CRC stage at diagnosis**  Localized  Regional  Metastatic  Unknown |  | 74 (34.4)  22 (10.2)  47 (21.9)  72 (33.5) |  | 998 (38.6)  481 (18.6)  550 (21.3)  554 (21.5) |  | 0.89 (0.74-1.08)  0.55 (0.37-0.82)  1.03 (0.79-1.34)  1.56 (1.28-1.91) |
| **CRC site**  Proximal colon  Distal colon  Rectum  Unspecified or more than one site |  | 119 (55.4)  38 (17.7)  35 (16.3)  23 (10.7) |  | 972 (37.6)  839 (32.5)  664 (25.7)  108 (4.2) |  | 1.47 (1.29-1.67)  0.54 (0.41-0.73)  0.63 (0.46-0.86)  2.56 (1.67-3.93) |
| **Supplementary Table 3, continued** |  |  |  |  |  |  |
| **CRC histology**  Adenocarcinoma  Polyp adenocarcinoma  Mucinous carcinoma  Signet ring  Neuroendocrine  Other histology^9^  Not histologically verified |  | 164 (76.3)  10 (4.7)  6 (2.8)  N/A  N/A  21 (9.8)  12 (5.6) |  | 2,170 (84.0)  161 (6.2)  92 (3.6)  9 (0.4)  17 (0.7)  62 (2.4)  72 (2.8) |  | 0.91 (0.84-0.98)  0.75 (0.40-1.39)  0.78 (0.35-1.79)  1.33 (0.17-10.49)  0.71 (0.09-5.29)  4.07 (2.53-5.54)  2.00 (1.10-3.63) |
| **CRC Surgery**  Colorectal surgeries^10^  No surgery |  | 190 (88.4)  25 (11.6) |  | 2.302 (89.1)  281 (10.9) |  | 0.99 (0.94-1.04)  1.07 (0.73-1.57) |
| **Mismatch repair status^11^**  MMR proficient  MMR deficient |  | 64 (53.8)  55 (46.2) |  | 991 (56.4)  767 (43.6) |  | 0.95 (0.80-1.13)  1.06 (0.87-1.30) |
| **Histologically verified polyps^12^**  Yes  No |  | 136 (63.3)  79 (36.7) |  | 731 (28.3)  1,852 (71.7) |  | 2.24 (1.98-2.52)  0.51 (0.43-0.61) |
|  |  |  |  |  |  |  |
| ^1^CRCs diagnosed 7-36 months after a negative colonoscopy.  ^2^CRCs diagnosed within 6 months after a preceding colonoscopy.  ^3^PRs and 95% CIs from robust Poisson regression testing associations of individual characteristics among T2D patients with PCCRC compared to T2D patients with dCRC.  ^4^IQR: interquartile range.  ^5^Recorded before or within 90 days after their first colonoscopy.  ^6^Time from initial T2D identification until CRC diagnosis.  ^7^Recorded before the date of PCCRC/dCRC diagnosis.  ^8^Charlson Comorbidity Index score: low = CCI score of 0; medium = CCI score of 1-2; high = CCI score of 3 or more.  ^9^ Including sarcomas, lymphomas, metastases, and unspecified histology.  ^10^Including total colectomy, partial colectomy, rectal resection, and other colorectal surgeries.  ^11^Restricted to CRCs with an available test for mismatch repair (MMR) status. CRCs demonstrating absent nuclear expression of one or more MMR proteins (MLH1, MSH2, MSH6, and PMS2) were considered MMR-deficient.  ^12^Histologically verified colorectal polyps (conventional adenomas and serrated polyps) or polypectomies recorded before the date of CRC diagnosis. | | | | | | |

| Supplementary Table 4**.** Number of deaths, survival probabilities, and hazard ratios (HRs) as a measure of mortality rate ratios in patients with diabetes mellitus type 2 (T2D) and colorectal cancer (CRC) categorized as post-colonoscopy CRC (PCCRC)^1^ or detected CRC (dCRC)^2^, Denmark, 2005-2015. Numbers <5 marked with “N/A” to ensure anonymity. | | | | | | | | | | | |
| --- | --- | --- | --- | --- | --- | --- | --- | --- | --- | --- | --- |
| **First year after CRC diagnosis** | | | | | | | | | | | |
|  | **PCCRC** | |  | **dCRC** | |  |  |  |  |  |  |
|  | **No. of deaths** | **1 year survival, % (95% CI)** |  | **No. of deaths** | **1 year survival, % (95% CI)** |  | **Crude HR**  **(95 % CI)** |  | **Adjusted HR^3^**  **(95% CI)** |  | **Adjusted HR^4^**  **(95% CI)** |
| **Total**  Male  Female | **77**  43  34 | **62 (54-68)**  59 (49-68)  64 (53-73) |  | **528**  306  222 | **78 (77-80)**  80 (78-82)  76 (73-79) |  | **2.04 (1.60-2.58)**  2.33 (1.69-3.21)  1.70 (1.18-2.43) |  | **1.98 (1.55-2.51)**  2.31 (1.68-3.18)  1.70 (1.18-2.44) |  | **1.89 (1.49-2.40)**  2.15 (1.56-2.98)  1.67 (1.16-2.40) |
| **Age at CRC diagnosis, y**  0-59  60-69  70+ | N/A  N/A  62 | 82 (44-95)  72 (57-83)  56 (48-64) |  | 33  102  393 | 83 (77-88)  85 (82-88)  74 (72-77) |  | 1.06 (0.25-4.42)  2.09 (1.17-3.72)  2.01 (1.54-2.63) |  | 1.09 (0.26-4.60)  2.08 (1.17-3.70)  2.02 (1.55-2.65) |  | 1.11 (2.26-4.65)  2.05 (1.15-3.67)  1.93 (1.48-2.53) |
| **Year of CRC diagnosis**  2005  2006-2010  2011-2015 | N/A  29  47 | 86 (33-98)  57 (45-68)  62 (53-70) |  | 33  208  287 | 69 (59-77)  76 (73-79)  80 (78-82) |  | 0.41 (0.06-2.99)  2.05 (1.39-3.02)  2.22 (1.63-3.03) |  | 0.52 (0.07-3.86)  2.01 (1.36-2.96)  2.12 (1.55-2.88) |  | 0.59 (0.078-4.40)  1.84 (1.25-2.72)  2.06 (1.51-2.81) |
| **T2D identification**  ICD code  Prescription  Both | 27  8  42 | 61 (48-71)  74 (54-86)  58 (48-67) |  | 187  62  279 | 74 (71-77)  80 (75-84)  80 (78-82) |  | 1.64 (1.10-2.46)  1.42 (0.68-2.97)  2.54 (1.83-3.51) |  | 1.60 (1.06-2.41)  1.53 (0.73-3.19)  2.55 (1.84-3.54) |  | 1.59 (1.06-2.39)  1.36 (0.64-2.89)  2.35 (1.69-3.26) |
| **T2D**  **duration, y**  <1  1-5  6-10  >10 | N/A  24  27  23 | 42 (6-77)  64 (51-74)  62 (50-72)  60 (46-72) |  | 96  167  140  125 | 78 (74-82)  79 (75-81)  78 (75-81)  77 (74-81) |  | 3.38 (1.07-10.68)  1.92 (1.25-2.93)  2.10 (1.39-3.17)  1.99 (1.27-3.10) |  | 3.20 (1.01-10.16)  1.93 (1.26-2.97)  2.04 (1.34-3.09)  1.81 (1.15-2.84) |  | 2.71 (0.84-8.68)  2.11 (1.37-3.25)  1.80 (1.18-2.74)  1.68 (1.07-2.64) |
| **Supplementary Table 4, continued** | | | | | | | | | | | |
| ^1^Colorectal cancer diagnosed 7-36 months after a negative colonoscopy.  ^2^Colorectal cancer diagnosed within 6 months after a preceding colonoscopy.  ^3^Adjusted for age, sex, and year of CRC diagnosis.  ^4^Adjusted for age, sex, year of CRC diagnosis, and CRC stage.  Abbreviations: hazard ratios (HRs); colorectal cancer (CRC); post-colonoscopy colorectal cancer (PCCRC); detected colorectal cancer (dCRC); confidence intervals (CIs). | | | | | | | | | | | |

| Supplementary Table 5**.** Number of deaths, survival probabilities, and hazard ratios (HRs) as a measure of mortality rate ratios in patients with diabetes mellitus type 2 (T2D) and colorectal cancer (CRC) categorized as post-colonoscopy CRC (PCCRC)^1^ or detected CRC (dCRC)^2^, Denmark, 2005-2015. Numbers <5 marked with “N/A” to ensure anonymity. | | | | | | | | | | | |
| --- | --- | --- | --- | --- | --- | --- | --- | --- | --- | --- | --- |
| **One to five years after CRC diagnosis** | | | | | | | | | | | |
|  | **PCCRC** | |  | **dCRC** | |  |  |  |  |  |  |
|  | **No. of deaths** | **1-5 years survival, %**  **(95% CI)** |  | **No. of deaths** | **1-5 years survival, %**  **(95% CI)** |  | **Crude HR**  **(95 % CI)** |  | **Adjusted HR^3^ (95% CI)** |  | **Adjusted HR^4^ (95% CI)** |
| **Total**  Male  Female | **47**  30  17 | **46 (35-57)**  38 (24-52)  56 (38-71) |  | **599**  402  197 | **53 (51-56)**  50 (47-54)  59 (54-63) |  | **1.33 (0.99-1.79)**  1.63 (1.23-2.37)  1.09 (0.66-1.78) |  | **1.35 (1.00-1.82)**  1.60 (1.10-2.31)  1.06 (0.65-1.75) |  | **1.43 (1.06-1.92)**  1.62 (1.12-2.35)  1.18 (0.72-1.95) |
| **Age at CRC diagnosis, years**  0-59  60-69  70+ | N/A  N/A  33 | 69 (21-91)  47 (25-67)  44 (30-57) |  | 44  185  370 | 60 (49-68)  54 (50-59)  52 (49-56) |  | 0.68 (0.16-2.80)  1.29 (0.72-2.31)  1.43 (1.00-2.05) |  | 0.63 (1.14-2.79)  1.26 (0.70-2.27)  1.44 (1.01-2.06) |  | 0.58 (0.13-2.59)  1.33 (0.74-2.39)  1.50 (1.05-2.15) |
| **Year of CRC diagnosis**  2005  2006-2010  2011-2015 | N/A  19  25 | 50 (11-80)  51 (35-66)  34 (16-54) |  | 24  255  320 | 67 (55-76)  61 (57-65)  28 (21-36) |  | 1.55 (0.47-5.14)  1.48 (0.93-2.36)  1.17 (0.78-1.76) |  | 1.47 (0.39-5.55)  1.52 (0.95-2.42)  1.21 (0.80-1.82) |  | 1.44 (0.37-5.62)  1.59 (1.00-2.55)  1.27 (0.84-1.90) |
| **T2D identification**  ICD code  Prescription  Both | 19  5  23 | 42 (24-59)  67 (36-85)  44 (27-59) |  | 189  70  340 | 56 (51-60)  57 (48-64)  51 (47-55) |  | 1.58 (0.99-2.54)  0.85 (0.34-2.12)  1.35 (0.89-2.06) |  | 1.60 (0.99-2.60)  0.76 (0.30-1.89)  1.43 (0.94-2.19) |  | 1.99 (1.22-3.25)  0.72 (0.29-1.81)  1.45 (0.96-2.22) |
| **T2D**  **duration, years**  <1  1-5  6-10  >10 | N/A  14  18  N/A | N/A  36 (23-49)  27 (15-40)  26 (13-42) |  | 110  167  182  140 | 47 (41-52)  48 (44-52)  34 (29-39)  35 (30-40) |  | 13.50 (3.20-57.0)  1.13 (0.65-1.95)  1.19 (0.73-1.93)  1.35 (0.76-2.39) |  | 14.35 (3.35-61.5)  1.20 (0.69-2.08)  1.21 (0.74-1.97)  1.30 (0.73-2.33) |  | 16.40 (3.78-71.1)  1.44 (0.83-2.51)  1.12 (0.69-1.84)  1.26 (0.70-2.27) |
| **Supplementary Table 5 Continued** | | | | | | | | | | | |
| ^1^Colorectal cancer diagnosed 7-36 months after a negative colonoscopy.  ^2^Colorectal cancer diagnosed within 6 months after a preceding colonoscopy.  ^3^Adjusted for age, sex, and year of CRC diagnosis.  ^4^Adjusted for age, sex, year of CRC diagnosis, and CRC stage.  Abbreviations: hazard ratios (HRs); colorectal cancer (CRC); post-colonoscopy colorectal cancer (PCCRC); detected colorectal cancer (dCRC); confidence intervals (CIs). | | | | | | | | | | | |

| Supplementary Table 6**:** Data Sources | |
| --- | --- |
|  | |
| **Registry** | **Description** |
|  |  |
| **Danish Civil Registration system (CRS)** | The CRS records data on vital status and migration for the entire Danish population. The unique 10-digit identifier (CPR) assigned to all legal residents at birth or upon immigration allows accurate and unambiguous linkage across all data sources at the individual level. Vital status is known to be coded essentially without errors in the Danish Civil Registration System, ensuring that information bias from this source is negligible.^1, 2^ |
|  | |
| **Danish Cancer Registry (DCR)** | The DCR contains information on all incident malignant neoplasms in Denmark since 1943. Cancers are coded according to the *International Classification of Diseases, Tenth Revision* (ICD-10).^3^ |
|  | |
| **Danish National Pathology Registry (DPR)** | The DPR contains information on all analyzed pathology samples in Denmark since 1997. Its underlying database, the Danish Pathology Data Bank, contains information on most biological samples dating back to the 1970s. The DPR uses the Danish version of the Systematized Nomenclature of Medicine (SNOMED) to store detailed pathology information.^4^ |
|  | |
| **Danish National Patient Registry (DNPR)** | The Danish National Patient Registry (DNPR) contains records of all surgical procedures performed in Denmark. It allowed us to identify and categorize persons in the CRC cohort as having or not having CRC surgery. The DNPR contains records on all in-hospital stays since 1977 and records on all hospital outpatient visits and contacts with emergency rooms since 1995. For each inpatient or outpatient contact, data include CPR number, dates of hospital admission and discharge, surgical procedures (including colonoscopies), and up to 20 discharge diagnoses coded according to ICD-8 until the end of 1993 and ICD-10 thereafter. Reporting of colonoscopies performed during outpatient visits has been mandatory since 1995.^5^ |
|  | |
| **Danish National Health Service Prescription Database (DNHSPD)** | The DNHSPD contains individual-level data on all prescriptions redeemed at Danish community pharmacies since 2004.^6^ |
| 1. Schmidt M, Pedersen L, Sørensen HT. The Danish Civil Registration System as a tool in epidemiology. Eur J Epidemiol 2014;29:541-9.  2. Pedersen CB. The Danish Civil Registration System. Scand J Public Health 2011;39:22-5.  3. Gjerstorff ML. The Danish Cancer Registry. Scand J Public Health 2011;39:42-5.  4. Erichsen R, Lash TL, Hamilton-Dutoit SJ, et al. Existing data sources for clinical epidemiology: the Danish National Pathology Registry and Data Bank. Clin Epidemiol 2010;2:51-6.  5. Schmidt M, Schmidt SA, Sandegaard JL, et al. The Danish National Patient Registry: a review of content, data quality, and research potential. Clin Epidemiol 2015;7:449-90.  6. Pottegård A, Schmidt SAJ, Wallach-Kildemoes H, et al. Data Resource Profile: The Danish National Prescription Registry. Int J Epidemiol 2017;46:798-798f. | |

| Supplementary Table 7**.** Registries and codes used in the analyses. | |
| --- | --- |
|  | |
| **Registry** | **Code** |
|  |  |
| **Danish National Patient Registry (DNPR)**  ICD-8 (1977-1993)  Chronic obstructive pulmonary disease  Atrial fibrillation/flutter  Cardiovascular diseases  Renal diseases  Alcohol-related diseases  Ulcerative colitis  Crohn’s disease  Diabetes mellitus  ICD-10 (since 1994)  Chronic obstructive pulmonary disease  Atrial fibrillation/flutter  Cardiovascular diseases  Renal diseases  Alcohol-related diseases  Ulcerative colitis  Crohn’s disease  Diabetes mellitus  Procedure codes  Before 1996:  Colonoscopy  NOMESCO (since 1996):  Colonoscopy  Polypectomy  Total colectomy  Partial colectomy  Rectal resection  Other colorectal surgeries | 491.00, 492.00  427.93  393-398, 400-404, 410-414, 427.09, 427.10, 427.19  403, 404, 580-583, 584, 590.09, 593.19, 753.10, 753.19, 792  291, 303, 577.10, 571.09, 571.10  563.19, 569.04  563.01  249.00, 249.06, 249.07, 249.09, 250.00, 250.06, 250.07, 250.09  DJ44  DI489  DI05-DI09, DI10-DI15, DI20-DI25, DI50  DI12, DI13, DN00-05, DN07, DN11, DN14, DN17-DN19, DQ61  DF10 (except DF100), DG312, DG621, DG721, DI426, DK292, DK860, DZ721  DK51  DK50  DE10-14, DO24 (except DO24.4), DG63.2, H36.0, DN08.3  910.70, 923.40, 923.60  KUJF32, KUJF35  KJFA15, KJGA05  KJFH  KJFB  KJGB  KJFA, KJGA |
|  | |
| **Danish Cancer Registry (DCR)**  ICD-10 codes  Colorectal cancer  Proximal colon cancer  Distal colon cancer  Unspecified or more than one site  TNM classification  Localized  Regional  Metastatic  Unknown | DC18, DC19, DC20  DC180, DC181, DC182, DC183, DC184  DC185, DC186, DC187  DC188, DC189, DC189  AZCD10-16, or AZCD19 + AZCD30 + AZCD40, AZCD10-14 + AZCD30 + AZCD49  AZCD10-13 + AZCD39 + AZCD40  AZCD10-16, or AZCD19 + AZCD31-33 + AZCD40  AZCD10-16, or AZCD19 + AZCD31-33 + AZCD41, AZCD10-16, or AZCD19 +AZCD30 + AZCD41, AZCD10-16, or AZCD19 +AZCD39 + AZCD41  AZCD13-16, or AZCD19 + AZCD39 + AZCD40 or AZCD49  AZCD15-16, or AZCD19 + AZCD30 + AZCD49 |
| **Supplementary Table 7, continued** | |
| **Danish National Pathology Registry (DPR)**  SNOMED codes  Colorectal polyps  Conventional adenomas  Serrated polyps  Dysplasia  Colorectal cancer histology  Adenocarcinoma  Polyp adenocarcinoma  Mucinous carcinoma  Signet cell cancer  Neuroendocrine cancer  Mismatch repair status  Mismatch repair deficiency  Mismatch repair proficiency | M82110, M82630, M82611, M8213F  M72040, M8213S, M82130  M72, M73, M74  M81403, M81433, M81443, M81453, M814F3, M82313, M82603  M82103, M82133, M82104, M82106  M84801, M82133, M82104, M82106  M84903  M82401, M82403, M82404, M82404, M82406, M82407, M82461, M82463  F29715, F29725, F29765, F29785, F297A5, F297B5, FE14A3  F29701, F29711, F29721, F29761, F29781, F297A1, F297B1, FE14A1  All SNOMED codes were required to be recorded in combination with a topography code indicating colon (T67) or rectum (T68). |
|  | |
| **Danish National Health Service Prescription Database (DNHSPD)**  ATC codes  Insulin  Metfomin  Sulfonylureas  Any other antidiabetic drugs | A10A  A10BA02  A10BB, A10BC  A10 without A10A, A10BA02, A10BB, A10BC |

Abbreviations: NOMESCO, Nordic Medico-Statistical Committee; SNOMED, Systemized Nomenclature of Medicine; ATC, Anatomic Therapeutic Chemical Classification

| Supplementary Table 8**.** Modified^1^ Charlson Comorbidity Index (CCI) codes and scores. Distribution of covariates among patients with diabetes mellitus type 2 (T2D) and colorectal cancer (CRC), categorized as post-colonoscopy CRC (PCCRC)^2^ or detected CRC (dCRC)^3^. Numbers <5 marked with “N/A” to ensure anonymity. | | | | | |
| --- | --- | --- | --- | --- | --- |
| **Covariate** | **ICD-8^4^ codes** | **ICD-10^5^ codes** | **CCI score** | **PCCRC, n (%)** | **dCRC, n (%)** |
| Myocardial infarction | 410 | I21, I22, I23 | 1 | 44 (17.6) | 318 (11.2) |
| Congestive heart failure | 427.09, 427.10, 427.11, 427.19, 428.99, 782.49 | I50, I11.0, I13.0, I13.2 | 1 | 43 (17.2) | 306 (10.8) |
| Peripheral vascular disease | 440, 441, 442, 443, 444, 445 | I70, I71, I72, I73, I74, I77 | 1 | 38 (15.2) | 312 (11.0) |
| Cerebrovascular disease | 430–438 | I60–I69, G45, G46 | 1 | 62 (25.0) | 454 (16.0) |
| Dementia | 290.09–290.19, 293.09 | F00–F03, F05.1, G30 | 1 | 6 (2.4) | 31 (1.1) |
| Chronic pulmonary disease | 490–493, 515–518 | J40–J47, J60–J67, J68.4, J70.1, J70.3, J84.1, J92.0, J96.1, J98.2, J98.3 | 1 | 43 (17.2) | 342 (12.1) |
| Connective tissue disease | 712, 716, 734, 446, 135.99 | M05, M06, M08, M09, M30, M31, M32, M33, M34, M35, M36, D86 | 1 | 17 (6.8) | 127 (4.5) |
| Ulcer disease | 530.91, 530.98, 531–534 | K22.1, K25–K28 | 1 | 23 (9.2) | 225 (7.9) |
| Mild liver disease | 571, 573.01, 573.04 | B18, K70.0–K70.3, K70.9, K71, K73, K74, K76.0 | 1 | 10 (4.0) | 68 (2.4) |
| Hemiplegia | 344 | G81, G82 | 1 | N/A | 11 (0.4) |
| Moderate to severe renal disease | 403, 404, 580–583, 584, 590.09, 593.19, 753.10–753.19, 792 | 12, I13, N00–N05, N07, N11, N14, N17–N19, Q61 | 2 | 21 (8.4) | 151 (5.3) |
| Any tumor | 140–152, 155-194 | C00–C75 (except C18 + C20) | 2 | 46 (18.4) | 499 (17.6) |
| Leukemia | 204–207 | C91–C95 | 2 | N/A | 15 (0.5) |
| Lymphoma | 200–203, 275.59 | C81–C85, C88, C90, C96 | 2 | 15 (6.0) | N/A |
| Moderate to severe liver disease | 070.00, 070.02, 070.04, 070.06, 070.08, 573.00, 456.00–456.09 | B15.0, B16.0, B16.2, B19.0, K70.4, K72, K76.6, I85 | 3 | N/A | 23 (0.8) |
| Metastatic solid tumor | 195–198, 199 | C76–C80 | 6 | N/A | 23 (0.8) |
| AIDS | 079.83 | B21–B24 | 6 | N/A | N/A |
| ^1^Diabetes codes and CRC codes are excluded, as they are not considered as covariates in this study.  ^2^CRCs diagnosed 7-36 months after a negative colonoscopy.  ^3^CRCs diagnosed within 6 months after a preceding colonoscopy.  ^4^ICD-8: *International Classification of Diseases, Eighth Revision* (1977-1993)  ^5^ICD-10: *International Classification of Diseases, Tenth Revision* (1994-) | | | | | |
